# Editorial: Biomass Modification, Characterization, and Process Monitoring Analytics to Support Biofuel and Biomaterial Production

**DOI:** 10.3389/fbioe.2016.00025

**Published:** 2016-03-10

**Authors:** Jason Lupoi, Blake Simmons, Robert Henry

**Affiliations:** ^1^Joint BioEnergy Institute, Emeryville, CA, USA; ^2^University of Queensland, Brisbane, QLD, Australia; ^3^Queensland Alliance for Agriculture and Food Innovation, Brisbane, QLD, Australia; ^4^Sandia National Laboratory, Livermore, CA, USA

**Keywords:** high-throughput screening assays, genetic manipulation, glycoside hydrolases, Raman spectroscopy, NIMS, cell-wall degrading enzymes, biomass pretreatment and fractionation

**The Editorial on the Research Topic**

**Biomass Modification, Characterization, and Process Monitoring Analytics to Support Biofuel and Biomaterial production**

This Frontiers Research Topic journeys through various challenges facing researchers seeking to develop fuels and products derived from lignocellulosic biomass. These challenges include: the rapid quantification of plant cell wall chemistry, enabling yields of potential monomeric sugars to be assessed, identification of plants possessing ideal trait that can be brought to the forefront of research efforts; once the native plant chemistry is known, how can yields be improved by chemically or genetically altering plant cell walls to reduce recalcitrance; does genetic modification of plants to increase accessibility to saccharification enzymes hinder the plant’s growth and/or function; are the innovative methods identified by researchers cost-effective and scalable to a commercial level? These topics are a sampling of the obstacles researchers combat when nominating a specific plant for downstream applications or implementing new deconstruction, genetic, or measurement strategies. How well can different plants can be broken down into useful, downstream precursor molecules? Efforts to develop a lucid picture of the chemical composition of abundant, diverse plants being explored as potential starting feedstocks has resulted in the evolution of high-throughput techniques that permit many more samples to be screened in much shorter periods of time. Advances in thermochemical and spectroscopic techniques have enabled the screening of thousands of plants for different phenotypes, such as cell-wall composition and monomeric sugar release. Some instrumental methods have been coupled with multivariate analysis, providing elegant chemometric predictive models enabling the accelerated identification of potential feedstocks. Rapid instrumental techniques have been developed for real-time monitoring of diverse processes, such as the efficacy of specific pretreatment strategies, or downstream products, such as biofuels and biomaterials. Real-time process monitoring techniques are needed for all stages of the feedstocks-to-biofuels conversion process to maximize efficiency and lower costs by monitoring and optimizing performance. These approaches allow researchers to adjust experimental conditions during, rather than at the conclusion, of processes, thereby decreasing overhead expenses.

The article in this book entitled “*Evaluating Lignocellulosic Biomass, Its Derivatives, and Downstream Products with Raman Spectroscopy*” illustrates how advances in Raman instrumentation, and the use of multivariate analysis has aided researchers in developing efficient, rapid methods to quickly evaluate diverse varieties of plants. Another hot research topic is how can we make better, more efficient enzymes beyond what may be naturally available? Just as high-throughput and automated methods are desirable to partition a diverse portfolio of potential feedstocks, gaging enzymatic performance and efficiency presents similar challenges that can be alleviated by employing techniques such as that illustrated in the two articles “*Use of Nanostructure-Initiator Mass Spectrometry to Deduce Selectivity of Reaction in Glycoside Hydrolases*” and “*Development of a High Throughput Platform for Screening Glycoside Hydrolases Based on Oxime-NIMS*”. Studies like these have probed a variety of phenotypic traits, including the assessment of dominant plant cell wall chemical constituents (glucan, xylan, lignin, etc.), and the quantification of products from enzymatically deconstructing the plants into simpler, molecular building blocks. “*Immunological Approaches to Biomass Characterization and Utilization*” reviews recent advances in the use of glycan-directed probes to evaluate plant cell walls, including glycan-directed monoclonal antibodies, in efforts to understand cell-wall composition and structure, and how manipulations to these affect the biosynthetic processes. Assessing the study and function of proteins, called proteomics, is typically throughput limited. “*Standard Flow Liquid Chromatography for Shotgun Proteomics in Bioenergy Research*” validates the standard flow technique, and shows how 800–1000 different proteins could be identified in complex samples. Pyrolysis of plants to form bio-oil presents an interesting option for the synthesis of fossil fuel replacements. Before these oils can be utilized, they must be fully characterized. How will the intrinsic biomass chemistry affect the resultant bio-oil? The article entitled “*Relationships between Biomass Composition and Liquid Products formed via Pyrolysis*” explores this topic.

These important traits can aid in gaging biomass recalcitrance, enabling researchers to devise appropriate deconstruction strategies. The article “*Optimization of Alkaline and Dilute Acid Pretreatment of Agave Bagasse by Response Surface Methodology*” describes a technique for optimizing the deconstruction of agave cell walls to maximize the total reducing sugars produced during saccharification. Various techniques used to combat biomass recalcitrance in eucalypts have been outlined in the article “*Efficient Eucalypt Cell Wall Deconstruction and Conversion for Sustainable Lignocellulosic Biofuels*”. Plants with high polysaccharide and lower lignin contents are known to breakdown to monomeric sugars more readily, but what happens if a plant does not naturally possess the ideal traits for biofuel production, like high cellulose and low lignin? Researchers can purposely tailor the plant’s chemical composition to be more favorable for conversion to sugars, fuels, or other value-added products. In “*Potential for Genetic Improvement of Sugarcane as a Source of Biomass for Biofuels*”, different strategies for improving the degradability of sugarcane are reviewed. Increases in the total biomass yield, cellulose content, or saccharification efficiency through, for example, reductions in lignin content, are pathways being evaluated to genetically improve plants as feedstocks for biofuels and bio-based chemicals. The biological pathways with which the plant cell wall is fabricated can shed light on potential genetic manipulations that can favorably alter the cell wall chemistry. The articles “*Phenotypic Changes in Transgenic Tobacco Plants Overexpressing Vacuole-Targeted Thermotoga maritima BglB Related to Elevated Levels of Liberated Hormones*” and “*Identification and Molecular Characterization of the Switchgrass AP2/ERF Transcription Factor Superfamily, and Overexpression of PvERF001 for Improvement of Biomass Characteristics for Biofuel*” illustrate this technique. Although plants have been previously domesticated for food and fiber production, the collection of phenotypic traits prerequisite for biofuel production may necessitate new genetic breeding schemes.

Lastly, the economic and social ramifications of using lignocellulosic biomass, such as the food versus fuel conflict, have been well documented. Can marginal lands provide us with the land required to achieve our energy needs in a cost-effective way? Cost-effective enough that we can seriously consider supplanting some fossil fuel usage on a much larger scale? This Research Topic includes a book review “*Book Review: Socio-Economic Impacts of Bioenergy Production*” of the recent Springer publication *Socio-economic impacts of bioenergy production*.

This collection of papers demonstrates how advances at multiple levels are likely to contribute to the successful industrial scale production of biofuels and biomaterials. Innovations in biomass composition have a major role to play in facilitating conversion and appropriate analytical methodologies are fundamental to managing the industrial process and guiding research and development.

## Author Contributions

JL wrote the editorial for this research topic. BS and RH critically read and edited the document.

## Conflict of Interest Statement

The authors declare that the research was conducted in the absence of any commercial or financial relationships that could be construed as a potential conflict of interest.

